# High resolution temporal transcriptomics of mouse embryoid body development reveals complex expression dynamics of coding and noncoding loci

**DOI:** 10.1038/s41598-017-06110-5

**Published:** 2017-07-27

**Authors:** Brian S. Gloss, Bethany Signal, Seth W. Cheetham, Franziska Gruhl, Dominik C. Kaczorowski, Andrew C. Perkins, Marcel E. Dinger

**Affiliations:** 1 Garvan Institute of Medical Research, Sydney, Australia; 2 St Vincents Clinical School, Faculty of Medicine, UNSW, Sydney, Australia; 3 The Gurdon Institute and Department of Physiology, Development, and Neuroscience, University of Cambridge, Cambridge, United Kingdom; 4 Center for Integrative Genomics, University of Lausanne, Lausanne, Switzerland; 5 Mater–UQ Research Institute, The University of Queensland, Translational Research Institute, Brisbane, Australia

## Abstract

Cellular responses to stimuli are rapid and continuous and yet the vast majority of investigations of transcriptional responses during developmental transitions typically use long interval time courses; limiting the available interpretive power. Moreover, such experiments typically focus on protein-coding transcripts, ignoring the important impact of long noncoding RNAs. We therefore evaluated coding and noncoding expression dynamics at unprecedented temporal resolution (6-hourly) in differentiating mouse embryonic stem cells and report new insight into molecular processes and genome organization. We present a highly resolved differentiation cascade that exhibits coding and noncoding transcriptional alterations, transcription factor network interactions and alternative splicing events, little of which can be resolved by long-interval developmental time-courses. We describe novel short lived and cycling patterns of gene expression and dissect temporally ordered gene expression changes in response to transcription factors. We elucidate patterns in gene co-expression across the genome, describe asynchronous transcription at bidirectional promoters and functionally annotate known and novel regulatory lncRNAs. These findings highlight the complex and dynamic molecular events underlying mammalian differentiation that can only be observed though a temporally resolved time course.

## Introduction

Over the past decade, transcriptomic investigations into the of nature embryonic stem cell (ESC) differentiation have elucidated key biochemical features of stemness and differentiation. Increasingly, it has become apparent that understanding the dynamics and coordination of gene expression signatures over time during the key phases of differentiation is critical to adequate characterization of fundamental biological processes.

Mouse ESC differentiation is a highly complex cascade of gene expression changes that allow single pluripotent cells in culture to progress to an organoid composed of cells reflecting three germ lineages within only five days. The spontaneous differentiation of these cells in culture has provided key insights into the developmental processes underlying the generation of the primary germ cell layers^1^. Microarray and RNA sequencing have provided a means to characterize the molecular transitions in gene expression underlying ESC biology and more recently single cell transcriptomic studies have provided the first glimpses into the molecular history of these cells^2^. However, it is clear that much more of the transcriptional landscape of ESC remains to be elucidated^3^.

Access to new technologies, such as massively parallel sequencing (MPS), has led to a dramatic increase in our knowledge of the mammalian transcriptome. Early genomic tiling array analysis indicated that most of the genome was transcribed into RNA^4^. MPS of the transcriptome validated this observation and revealed that the majority of the mammalian genome is pervasively transcribed as interlaced and overlapping RNAs^5^, many of which lack protein-coding potential^6^. The large number of long-noncoding transcripts (lncRNA) has become the focus of significant interest due to their exquisite cell type specific expression^7^, potent biological function^8, 9^, and rapid transactivation of cellular processes^10^. However, in general, lncRNAs are lowly expressed and short lived^11^, possibly because, unlike mRNAs that require translation, are able to exert their function directly. These qualities obfuscate their identification and characterization with traditional approaches that are tuned to the properties of mRNAs^12^. Owing to the relative infancy of the field, the vast majority of noncoding transcripts are of unknown function^13^. Additionally, the expression patterns of these genes imply that their function is dependent on cellular context and likely regulatory^8^, thus the identification of these molecules and the context in which they act remains a research priority^14^.

Various expression profiling studies, using both microarrays and RNA-seq^15–18^, have been used to explore the molecular changes occurring during ES cell development, typically at 24-hourly or more. This potentially has lead to incomplete gene expression relationships through the phenomenon of temporal aggregation bias whereby each time point is assumed to represent all the signaling changes occurring in that time window^19^. In contrast to single cell based approaches- which provide insight into the state of individual cells - examinations of whole cell populations provides system-wide behavior and a practical means to explore gene expression dynamics across time. The combination of these techniques has recently shed light on the molecular framework of cellular differentiation^20^. Higher temporal resolution has also shown rapid induction (within two hours of retinoic acid stimulation) of lncRNAs associated with the HOX locus^21^. Furthermore, high temporal resolution has provided valuable insights into transcriptional annotation and regulation in drosophila^22, 23^, Xenopus^24^ and C.elegans^25^.

Here we show that additional temporal resolution of the global transcriptome in spontaneously differentiating mESC cells following LIF withdrawal enables the capture of the rapid and complex dynamic regulatory and noncoding changes occurring during ES development. We analyzed the transcriptome of differentiating mouse ESCs at six-hourly intervals over a five-day period, over which time the three primordial germ layers are specified. Using this fine-resolution temporal sampling approach, we identify significant transitions in the transcriptome and large-scale shifts in observable transcription factor activities that could not be observed at 24 hourly sampling periods. Moreover, we identify entirely novel coding and noncoding transcripts that are expressed only within specific sub-24-hour window. By leveraging the high sampling frequency of the data, we are able to both accurately recapitulate known regulatory cascades in ES development and predict and refine others. Finally, using correlative approaches, we can infer functions for uncharacterized lncRNAs and predict the regulatory centers across the genome that coordinate early development.

## Results

### The dynamic transcriptome of mESC differentiation at high temporal resolution

**Figure 1:**
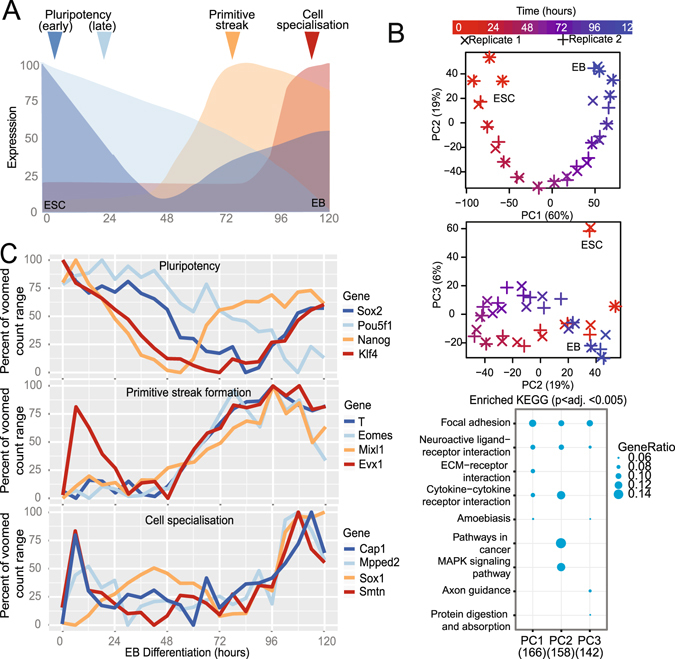
Global and gene-specific evaluation of augmented temporal resolution in mES differentiation. (**A**) Schematic of mouse embryonic stem cell (ESC) differentiation into embryoid bodies (EB) over the time course evaluated here. (**B**) Analysis of the top three principle components (PCs) based on the 2,000 most variable genes from biological duplicate-6 hourly transcriptomes and KEGG pathway enrichment for 500 genes contributing most to each of the top three PCs. (**C)** Expression profiles of genes associated with pluripotency, primitive streak formation and cell specialization.

A median 42-million, paired-end 100-bp reads (Supplementary Fig. S1A) were mapped from stranded, poly-A derived cDNA libraries derived from biological duplicate, six-hourly time courses of mESC differentiation over five days where key differentiation programs occur (0–120 hours, Fig. 1A). Transcript-level expression data was generated as previously described^26^, then normalized for library size and transformed for data visualization and differential gene expression analysis. Evaluation of 24 hourly time points indicated that our data was comparable to previously published data in a similar model^27^ (Supplementary Fig. S1B). An interactive gene expression portal was created to visualise this data (https://betsig.shinyapps.io/paper_plots).

To assess the reproducibility and provide confidence in the biological validity of the global transcriptome trends, a principle components analysis (PCA) was performed on the 2,000 most variable genes (Fig. 1B). This analysis indicated that biological replicates clustered closely, indicating that synchrony was retained, and that the major contributor to the determination of variance was explained by time. Deconvolution of the dimensions yielded time-dependent expression (in the first dimension) of genes enriched in focal adhesion/ECM interactions KEGG pathways. Interestingly, the second dimension deconvolution (PC2), in which undifferentiated ESCs resemble the more differentiated embryoblast, yielded genes enriched in MAPK-signaling and cancer pathways, implying that the process of differentiation involves a partial reacquisition of a mitogenic signaling. In the third component (PC3), in which the undifferentiated ES cell is separate, the axon-guidance pathway was enriched. We then evaluated expression patterns of genes associated with pluripotency, primitive streak formation and cell specialization (Fig. 1C). We observed that, although the gene expression patterns were broadly consistent with published studies (Supplementary Fig. S1B), there were changes in expression on less than 24 hourly timeframes that could not be attributed to stochastic expression changes (within the top 5% of deviation of all genes from expression values loess-smoothed over 24 hours). Similarly, we observed that no single 24-hourly measure was representative of the average expression over that day (Mann-Whitney U p. adj. <<<0.0001) and that more than 1,000 genes displayed a more than 2-fold difference mostly in the first 24 hours of differentiation (Supplementary Fig. 1C,D). Therefore we conclude that 24-houly expression profiles are unable to capture the intervening expression changes and that 6-hourly measurements reduce the phenomenon of temporal aggregation bias^19^, providing enhanced resolution of transcriptional changes in this system.

To evaluate characteristics of sub-24 hour gene expression patterns in in the transcriptome of developing ESCs, we observed that, compared to 24 hour time points, 417 more genes had counts data considered sufficient for differential gene expression analysis (>1 CPM in at least two samples); this was associated with a relative increase in detected noncoding genes (13% (588 vs. 520); defined as antisense, lincRNA and processed transcript biotypes) over protein coding, (2% (13336 vs. 13036) despite being underrepresented in the total pool (chi-squared p value < 0.001, Supplementary Table S1 and Supplementary Fig. S1E)). The additional time points allowed the assembly of 58% more novel multiexonic intergenic, antisense and intronic noncoding RNAs from the data - indicating that a substantial proportion of noncoding transcripts are present on timescales much shorter than 24 hours. These results indicate that enhanced temporal resolution reduces the phenomenon of temporal aggregation bias and allows the observation of more distinct cell expression states than typical time-courses.

### An improved signaling cascade described by higher temporal resolution

**Figure 2:**
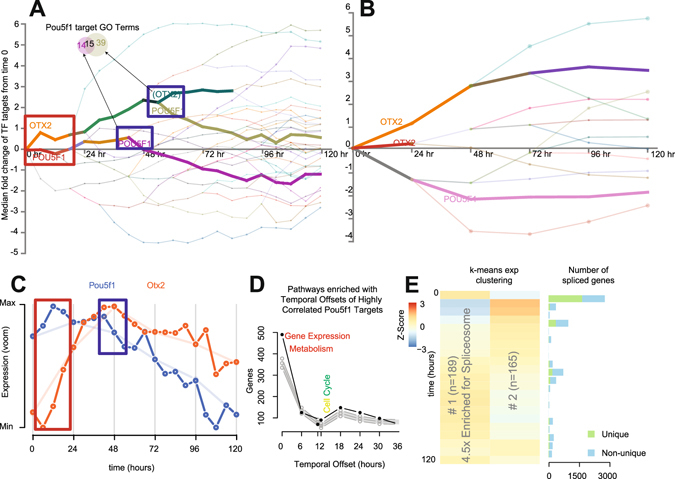
Insights into regulatory and gene expression kinetics. (**A** and **B**) Observable regulatory network dynamics at 24- and 6-hourly measures with Otx2 and Pou5f1 target containing profiles annotated and in bold, See Supplementary Fig. S2 for full figure. Transcriptomes at 24- (top) and 6-hourly (bottom) were subjected to DREM analysis of mouse TF/target gene interactions. Lines represent the median fold change (relative to time 0) of grouped TF target genes- representing activity of the TF itself, line colors are assigned by branch and are not comparable between panels. A p-value cutoff of 0.001 was applied to calculating divergent TF activity (splits). (**C**) Expression of the key transcription factors *Pou5F1* (*Oct4*) and *Otx2*. Red and blue boxes correspond to the time points highlighted in part A. (**D)** Distribution of the number of genes and the time delay required to meet a maximum correlation (>0.8) between gene targets of Pou5f1 and *Pou5f1* itself compared to 95% quantiles of 500 random gene selections. (**E)** Two k-means clusters of short-lived RNA (slRNA) genes displaying differential expression without changes at 24-hourly time points (adj. p < 0.0001).

Increased sampling frequency can provide a powerful insight into understanding of the contribution of gene regulatory networks to cellular differentiation^22^. We utilized the DREM v2 analysis tool^28^ to evaluate transcription factor (TF) target gene expression patterns. Divergence of gene targets responsive to groups of TF at each time point, either 24-hourly or 6-hourly (Fig. 2A,B) was shown if the overall difference was significant at p < 0.001. Compared to 24-hourly, the observed complexity was significantly higher, especially in the first 48 hours. We observed that significant changes in gene regulation occurred continuously within the 24-hour windows. Most notably, first 24 hours following depart from pluripotency resembles an ordered cascade of TF activity (Figs 2A and S2A) with large-scale changes in TF activity at 12, 18 and 24 hours; of which little can be deduced measuring at just 24 hours (Figs 2B and S2B). Focusing on the interplay between two key transcription factors (Otx2 and Pou5f1/Oct4^29^, Fig. 2A), we observed a rapid rise in Otx2 activity in the first six hours and stable Pou5f1activity for the first 24 hours (Red Box). Otx2 activity did not coincide with mRNA expression of the factor itself (Fig. 2A vs. C), although previous studies have observed increased in Otx2 protein expression within 3-hours of differentiation^29^, however periodic drops in *Pou5f1* mRNA expression appeared to coincide with decreases in POU5F1 target genes, we calculated the time taken for *Pou5f1* expression to result in changes in highly positively correlated (r > 0.8) target genes using a cross-correlation approach similar to ref. 30. We then evaluated how these “delays” enriched for certain Reactome pathways (Fig. 2D). We found rapid effects for targets enriched for “gene expression”- and a delayed effect on “cell cycle” pathways compared to a null distribution produced by 500 random “target” selections (grey). These were similarly observed in the DREM GO-term enrichment tool for Pou5F1 targets decreasing in expression at 42 (early- Transcription Factor Activity) and 54 hours (late- Epithelial Proliferation; Fig. 2A, Blue Box & Supplementary Fig. S2C) and associated with the decrease in *Pou5F1* expression (Fig. 2C, Blue Box). Importantly, Pou5F1 mRNA and protein expression are temporally correlated^29^. This result implies that TF-target genes may be activated in an ordered- time dependent fashion. To explore this more broadly, we evaluated other TF-target gene temporal dynamics for other TFs that exhibited strong positive or negative correlations between the TF and their target genes. We found evidence of highly structured TF-target expression patterns in time for negatively correlated Pou5f1 and Suz12 targets, as well as positively correlated Nanog, Myc, Sox2 and Suz12 targets (Supplementary Fig. S3).

These observations of precise temporal ordering of transcriptional events emphasize the importance of factoring time delays into understanding gene regulatory networks^31^. This highlights the capacity of increased temporal resolution to directly identify regulator-target gene interactions instead of relying on inference; which is common in cross-correlation approaches.

### Increased temporal resolution identifies genes with previously uncharacterized expression patterns (Short-lived (slRNA) & Cycling (cycRNA))

Having established that the increased temporal resolution markedly improves the molecular framework for evaluating the contribution of gene expression to ES differentiation, we next sought to identify gene expression signatures previously unable to be resolved using lower temporal resolution. For each 24-hour period, we identified genes that were differentially expressed between 0 and 6, 12 and 18 hours but not between any 24-hourly measures (Supplementary Fig. S2D). We identified 1,135 genes with significant changes in gene expression that were unchanged between any 24-hourly comparison (adjusted p < 0.0001). Of these, 354 were differentially expressed for more than half of the corresponding 24-hour window, mostly in the first and last 24-hour periods. These genes were described as short-lived RNAs (slRNAs). slRNA expression patterns over the first 24 hours of differentiation were found to be positively correlated with the same time window of retinoic acid directed differentiation^21^ (Supplementary Fig. S2E) implying that these genes may form part of the early response to differentiation signals. K-means clustering and KEGG pathway analysis of the expression profiles of these genes (Fig. 2E) revealed enrichment in genes associated with the spliceosome (adjusted p < 0.05) dramatically decreasing in expression over the first 24 hours before returning slowly to baseline. To examine whether this impacted gene-splicing patterns, we employed a differential exon (DEX) analysis between consecutive six-hourly time points and counted the number of genes displaying DEX usage (adjusted p value < 0.01 Fig. 2E). Consistent with previous studies, the alternate splicing was most highly associated with cell differentiation^32^ (Fig. 2E). Increased temporal resolution has elucidated that these changes happen very rapidly (majority of changes in the first six hours), and that slRNAs may be involved in suppressing the alternate splicing of genes and limiting transcriptional plasticity.

Some slRNAs appeared to have periodic expression profiles. We thus sought to uncover periodic expression patterns genome-wide, by applying a fast-Fourier transformation to our data (see Methods). Periodogram analysis was utilized to ascertain the dominant cycling period for each gene. We found 137 genes, which we termed cycling RNAs (cycRNAs), sharing the same dominant cycling period of less than 36 hours in both biological replicate experiments (Supplementary Table S2). Supporting the efficacy of the approach, we found *Clock*, which encodes a key regulator of circadian rhythm in mammals, to have a period of 24.2 hours. We identified 20 genes that displayed characteristics of both slRNAs and cycRNAs (Supplementary Fig. S2F), including *Ewsr1* and *Clk1*, involved in gene splicing^33, 34^ as well as five uncharacterized lncRNAs. Given the highly specific expression patterns in this context, we propose these genes may similarly have roles in maintaining or establishing biological rhythms. Together these investigations show that the augmented temporal resolution approach provides access to gain insights from regulatory pathways by identifying transitions in expression that would otherwise have remained hidden.

### Increased temporal resolution gives insight into local gene regulation in the genome

**Figure 3:**
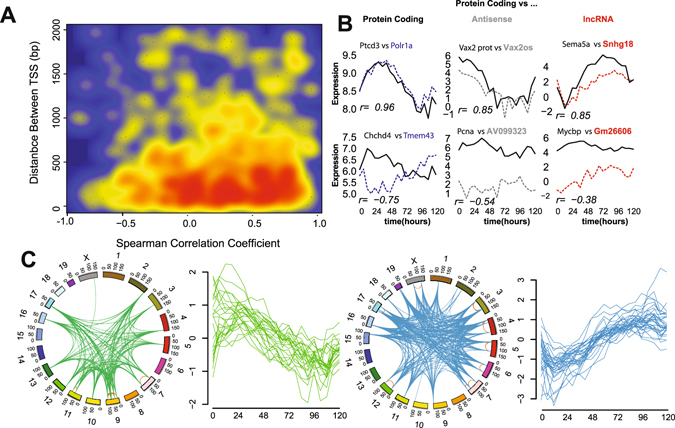
Analysis of gene co-expression patterns using augmented temporal resolution. (**A**) Smoothed scatter plot showing the correlation coefficient across the time course vs. distance between transcriptional start sites (TSS) of bidirectional gene pairs. Blue indicates no gene pairs; yellow and red indicate increasing numbers of pairs sharing similar properties. (**B**) Expression patterns of example bidirectional genes of the same or different gene biotype. Spearman’s correlation coefficient is reported for each pair. (**C**) Genomic location (circos) and expression pattern (line plot) of two independent co-expressed groups of 5 or more contiguous genes sharing correlated expression (r > 0.5).

Evaluating gene transcription at high temporal resolution in a highly dynamic process such as ES development, we anticipated that it might be feasible to dissect structural gene regulation within a given locus. To explore this possibility, we examined expression arising from transcripts that are oriented head-to-head as so-called bidirectional pairs^35, 36^. Interestingly, we observed that the antisense transcript for *Evx1* (Fig. 1C) displayed a previously unobserved^15^ increase in expression in the first 24 hours after departure from pluripotency that was reflected in its paired protein coding gene *Evx1* (Supplementary Fig. S4A), highlighting the increased power of frequent sampling over time. In total, we identified 1,251 gene pairs with bidirectional transcriptional start sites (TSS) within 2,000 bp and evaluated correlation coefficients across the time course, distance between TSS and median expression values. Consistent with other studies, we found expression correlation more positive for bidirectional gene pairs than random transcript pairs^35^ (Supplementary Fig. S4B). We were also able to show that the distance between TSS of highly correlated bidirectional gene promoters is typically less than 500 bp (Fig. 3A), consistent with a common regulatory domain. Highly correlated or anti-correlated genes pairs displayed differences in total gene expression, particularly with discordant gene biotypes (Fig. 3B). We found that protein coding gene pairs were more likely to be of similar expression levels and positively correlated (Mann-Whitney p < 0.05) than protein coding/noncoding pairs (Supplementary Fig. S4C). Applying a variant of the temporal offset analysis used to measure TF- gene target delays, we calculated the time taken and defined the apparent driver gene type for peak correlation in coding/noncoding bidirectional pairs (Supplementary Fig. S4D,E). This did not reveal a generalized bias in either time taken or particular “driving” gene type. However, this approach shows that the lncRNA *Hotairm1*, required for activation of Hoxa1^37^, appears to have a six-hour delay between its expression changes and HoxA1. We present evidence of other examples of lncRNA-led expression of protein coding genes in small numbers of bidirectional pairs (Supplementary Fig. S5).

To investigate whether the strong correlative potential between gene pairs could facilitate the identification of regions of the genome that are coordinately regulated^38^, we scanned across the genome for regions containing five or more contiguous genes that were co-expressed (r > 0.5). This revealed 59 regions with a mean size of 821 kb -each containing 5–14 genes (mean of 6) genes. To examine the higher order chromatin architecture of these regions, we compared these regions to published data on topological associated domains (TADs) for mouse ESCs^39^. We found that the majority of the regions were each contained within a single TAD (Supplementary Fig. S4F), increasing the likelihood for a common regulatory architecture. Evaluation of gene-expression patterns across these regions revealed evidence of high co-expression at both the inter- and intra-chromosomal levels (Supplementary Fig. S4G). We assembled a map of regions of the mouse genome displaying high levels of clustered co-expression (Fig. 3C) by comparing the expression profiles of the regions. Two independent modules were identified with distinct decreasing (green)- and increasing (blue) expression patterns with differentiation. Given the independent location and expression patterns of these clusters, we suggest they may form core expression-factories of cellular differentiation. In support of this notion, this analysis identified the a region -associated with the “increasing module”- containing the imprinting locus of *H19, Igf2, Tnn3* and *Mrpl23* ^40^ (Supplementary Fig. S4H); previously shown to be activated in concert during early stem cell differentiation^41^.

These investigations illustrate how analysis of high-resolution temporal transcriptomic data provides an independent and convenient approach (relying only RNA-Seq) to guide the partitioning of the genome into regulatory domains.

### Increased temporal resolution refines the noncoding landscape of mESC differentiation

Having shown that rapid changes in lncRNAs are a key feature of ES differentiation, and that co-expression analysis is a powerful tool for understanding gene regulation with augmented temporal resolution, we sought to unravel the roles that lncRNAs might play in ESC differentiation.

**Figure 4:**
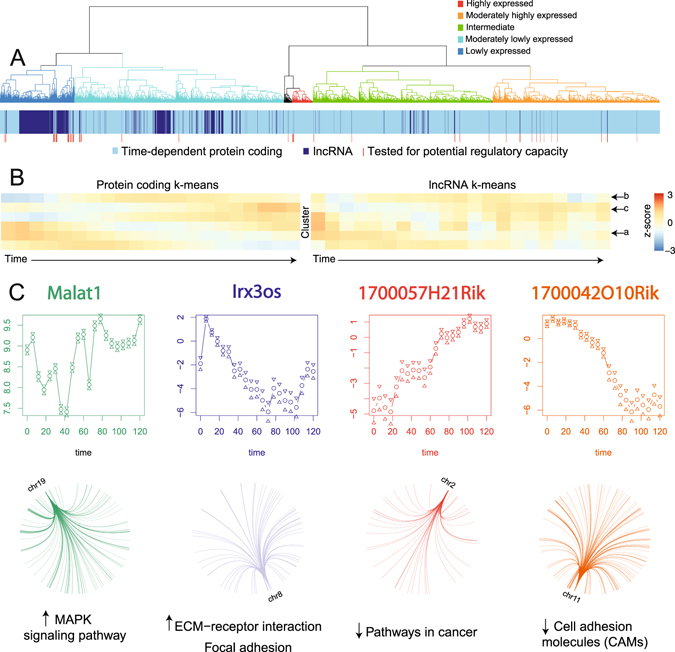
Augmented temporal resolution of ncRNA expression in cellular differentiation. (**A**) Hierarchical clustering of lncRNAs (dark blue) with time-dependent protein coding genes (light blue) by their expression patterns over time. Dendrogram was manually colored to reflect gene expression levels of the top-level clusters. (**B**) K-means clustered expression profiles of protein coding genes compared to the same number of lncRNA gene expression clusters. Common profiles are marked with arrows. (**C**) Expression profiles of four lncRNAs predicted to have regulatory roles in ES development as well as the genome location & pathways enriched in their gene targets. * Malat1* and *IRX3os* display a positive association with their targets, whereas *1700057H21Rik* and *1700042O10Rik* have a putative repressive impact.

Analysis of gene annotations yielded confident expression data for 588 lncRNA genes at six-hourly resolution (520 for 24-hourly, Supplementary Table S1). Indeed, added temporal resolution increased information of all noncoding transcript biotypes indicating that a proportion of these genes were only present for a short duration in this system. It is important to note that these do not represent the entirety of lncRNA expression in this process since the depth of sequencing was not standard for lncRNA coverage^42^ and the poly-A selection for cDNA would have missed enhancer RNAs, miRNA precursors and Nuclease P processed lncRNAs^43^. Clustering the observed lncRNA expression patterns with time-dependent protein coding gene expression showed that lncRNAs were enriched at lower expression levels and shared related expression profiles to protein coding genes (Fig. 4A). This relationship was further examined whereby K-means clustering of these expression profiles compared to clustering of a similar number of time-dependent protein coding genes (Figs 4B and S6A) revealed clusters of lncRNA genes resembling gene expression patterns associated with stemness (cluster a) primitive streak formation (cluster b) and WNT signaling (cluster c)^15^. As co-expression has been illustrated to provide valuable insight into lncRNA function^18^, the additional correlative strength afforded by this study is anticipated to more reliably guide the functional association of these lncRNAs with these processes. The dynamics of lncRNA expression observed here indicate that future studies using RNA capture sequencing or higher sequencing depth of Ribosome depleted RNA will provide more comprehensive insights into the role of lncRNA in the molecular events underlying cell differentiation.

As lncRNAs often exert their function through guiding or assembling transcriptional machinery, we sought to identify potential regulatory lncRNAs in this system. We selected 50 highly or variably expressed lncRNAs (Fig. 4A) and tested for evidence of gene regulatory behavior across the transcriptome. The temporal resolution allowed the use of time to resolve precedence, thus adding weight to a potential causal relationship. Since lncRNAs typically exert their function as a transcript, we set a maximum time offset of 18 hours to avoid secondary (altered protein level) effects and examined patterns in the predicted gene targets of lncRNAs (r > 0.8, divided by positive or negative associations). Reactome pathway analysis revealed that 11 of these lncRNAs (including well characterized lncRNAs, Supplementary Fig. S6B and C) were potentially involved in regulating networks of genes associated with key developmental processes (p.adj <0.05, Supplementary Fig. S6C). These analyses assigned target gene networks consistent with characterized lncRNA biological functions for *Malat1* (oncogenic)^44^, *Neat1* & *Rian* (association with gene repression)^45^ and *Meg3* (tumour suppressor)^46^. Interestingly, these data suggest that the pro-tumorigenic function of *Malat1* may be mediated through facilitating the increase of MAPK signaling molecules. Importantly, these data also provide testable evidence for seven previously uncharacterized lncRNAs role in ES development and describes a map of regulatory interactions potentially driven by lncRNAs (Fig. 4C) whereby lncRNAs expression may impact coding gene expression across the genome. The identification of lncRNAs with a predicted biological role is important for unraveling lncRNA function, providing candidate functional lncRNAs and providing a level of molecular detail that is currently lacking in many lncRNA studies.

## Discussion and Conclusions

Transcriptional regulation of key biological events is a key feature in understanding the complexity of cellular processes. Here we describe a detailed transcriptomic resource for research in cellular development, a framework for unraveling this detail and identifying new targets for analysis. We also present a comprehensively detailed survey of noncoding transcripts throughout early stem cell development. We have identified many previously uncharacterized noncoding RNAs with potentially pivotal roles in cellular differentiation. This will provide a valuable tool for researchers unraveling the transcriptional complexity of cellular differentiation.

### Increased interpretive power

The understanding of molecular events underlying the departure from pluripotency has been determined by the extant knowledge of how biological functions are exerted – often measured at 24 hourly or greater intervals. We hypothesized that interpretations of this model were missing detail in light of evidence indicating the unforeseen dynamics in RNA biology and regulation. By probing this detail with finer time distinctions, we show that gene expression profiles of well-characterized genes display significant variation of expression levels and that more detail can still be gleaned with increased sampling frequency (Supplementary Fig. S7A and B). Importantly, these variations are manifest in a significantly more complex gene regulatory framework. This is consistent with a reduction in temporal aggregation bias^19^ and highlights early array-based investigations in yeast demonstrating the importance of sufficient temporal resolution in understanding gene expression patterns^47^. As such, much detail is likely missing from other systems that involve a change in phenotype or cellular behavior. With large-scale transcriptomic analyses becoming increasingly accessible, it is opportune to revisit other well-studied transitions with the view of improving understanding and applicability of their results rather than relying on presuppositions about gene expression patterns^48^.

### Insights into short bursts of transcription

We have shown the benefit of frequent sampling over time in observing the transcription of genes that are observable only within sub-24 hourly windows. This approach highlights the importance of taking into account the presence of short-lived transcripts and shows that cells express more of the transcriptome in a time-dependent fashion. To this end, we have identified rapid changing and periodically expressed genes, which we term short-lived (slRNA) and cycling (cycRNA), that were unobservable outside this framework. That many slRNAs exhibited changes in expression over the first 24 hours of differentiation is consistent with rapid initial cellular response to stimuli^21, 49^. Indeed, it is likely that significant gene expression changes- especially noncoding- occur on timeframes shorter than those presented that may not be amenable to optimal time point prediction strategies^48^. By probing deeper into time-dependent gene transcription-possibly by interpolating available datasets-^47^ it will be possible to uncover further complexity underlying cellular plasticity and gene regulation. These observations reinforce the concept that adequate temporal resolution is vital for describing biological transitions- for example in dissecting primary from follow on effects in gene knockdown studies – and that end-point analysis likely does not reflect the complex biology of phenotype changes.

### Insight genome organization and regulation

Similarly, by using time to separate the order of gene transcription, we have been able to predict local gene regulation across the genome. We have been able to observe concerted gene expression (in *trans*) of hundreds of genes separated by large genome differences (in *cis*). Typical studies of this nature involve correlative analysis requiring large samples sizes and resources^50^. We have instead leveraged the time axis to achieve these as well as discriminate driver from passenger molecular events. This has allowed the estimation of the time delay for changes in expression of regulatory molecules to manifest in changes in their target gene transcription and we have been able to unravel a potentially complex network of gene profiles responding to lncRNA transcription. Finally, we have been able to use an integrated biological system to draw strong associations in trans relationships with bidirectional promoters. Typically these associations are observed by using thousands of gene expression profiles, yet here we have been able to do so with only 42 transcriptomes (duplicate time courses of 21 points each).

## Methods

### Sample Generation and Library Preparation

Biological duplicate, low passage number (P18) W9.5 ESCs were cultured and differentiated as described previously^15, 51^. Cultures were harvested every six hours from the induction of differentiation to 120 hours post differentiation induction. Total RNA from cultures was purified using Trizol (Life Technologies) and DNase treatment was performed by RQ1 DNase (Promega) according to the manufacturer’s instructions. RNA integrity was measured on a Bioanalyzer RNA Nano chip (Agilent). RNA-Seq library preparation and sequencing of Poly-A-NGS libraries generated from 500 ng total RNA using SureSelect Strand Specific RNA Library Preparation Kit (Agilent) were performed according to the manufacturer’s instructions at the same time to minimize batch effect. Paired-end libraries were sequenced to the first 100 bp on a HiSeq 2500 (Illumina) on High Output Mode.

### Quality control and read mapping

Library sequencing quality was determined using FastQC (Babraham Bioinformatics) and FastQ Screen (Babraham Bioinformatics). Illumina adaptor sequence and low quality read trimming (read pair removed if <20 base pairs) was performed using Trim Galore! (Babraham Bioinformatics: www.bioinformatics.babraham.ac.uk/). Tophat2^52^ was used to align reads to the December 2011 release of the mouse reference genome (mm10) as outlined by Anders *et al*.^26^. Read counts data corresponding to GENCODE vM2 transcript annotations were generated using HTSeq^53^. *de novo* transcript assembly was performed on each merged BAM file using Cufflinks’ reference annotation based transcript (RABT) assembly^54^, using the Gencode vM2 transcriptome^55^ as a guide (options: -u -I 500000 -j 1.0 -F 0.005-trim-3-dropoff-frac 0.05 -g gencode.vM2.annotation.gtf–library-type fr-firststrand). Transcript assemblies were then merged using Cuffmerge^56^ using default parameters, and compared to the Gencode vM2 reference transcriptome using Cuffcompare^56^. Novel transcripts with a Cuffcompare class code of j, i, o, u or x were filtered using three steps to find novel lncRNAs. First, a Browser Extensible Data (BED) format file was generated using a python script (https://gist.github.com/davidliwei/1155568) and any single exon transcripts were removed. Second, the FASTA-formatted sequence for each transcript was obtained using BEDTools^57^, the nucleotide (nt) length and open reading frame (ORF) size found using Perl scripts, and those with a length less than 200 nt or a ORF size greater than 300 nt were removed. Lastly, transcript sequences were submitted to Coding Potential Calculator (CPC)^58^, and those with a coding potential of >0 were removed.

### Bioinformatics

All analyses were performed in the R Statistical Environment^59^. Briefly, counts data were background corrected and normalized for library size using edgeR^60^, then transformed using voom^61^ for differential expression analysis using LIMMA^62^. Transcription Factor (TF) activity was inferred from gene expression data using DREM^28^ with a branching P-value of 0.001 based on curated TF-target gene lists associated with mouse ESC differentiation from ChEA^63^. TF-target gene was calculated by maximal Pearson’s correlation coefficient of >0.8 using a custom autocorrelation analysis and verified with the “ccf” function in R. Gene differential exon (DEX) usage was analyzed by DEXSeq^64^ on vM2 gene annotations using default settings and an adjusted p value cutoff of 0.001 for DEX between biological duplicates at each consecutive time-point. Genome position analyses were performed using genomic ranges^65^ based on vM2 annotations imported with ‘rtracklayer’^66^ and Pearson’s correlation coefficient of gene expression Bidirectional genes were defined as two genes with expression data on opposing strands with <2000 bp between the transcriptional start sites (TSS). Co-expressed gene clusters were defined as >5 contiguous genes with expression data displaying a Pearson’s Correlation Coefficient of >0.5 with neighbouring genes. Cluster co-expression data was visualized with corrplot^67^ and Cytoscape (v3.1.0)^68^, location of related clusters was visualized by Circos^69^. Gene expression periodicity was measured on 120 interpolated expression values^70^ for each replicate time series using GeneCycle^71^, candidate periodically expressed genes were identified as having the same calculated dominant cycling frequency between biological replicates. Time-dependent expression signatures were established using maSigPro^72^ with a replicate correlation coefficient cutoff of 0.8. Target genes of potential regulatory (top 50 most highly and/or variably expressed) lncRNAs were identified using the GeneReg package^73^ on 100 point-interpolated expression data based on fitted expression values between duplicates and setting a maximum time delay of 18 hours and a global correlation coefficient of 0.9 and visualized using Cytoscape. Gene lists were functionally annotated with KEGG and Reactome pathways (adjusted p value < 0.05) using the clusterProfiler and ReactomePA packages^74^.

### Availability of data and material

Data has been deposited into GEO under accession number GSE75028.

## Electronic supplementary material

Supplementary Figures and Tables
