# Supplementary material for: High resolution temporal transcriptomics of mouse embryoid body development reveals complex expression dynamics of coding and noncoding loci
Source: Sci Rep. 2017 Jul 27;7:6731. doi: 10.1038/s41598-017-06110-5 (PMC5532269; doi:10.1038/s41598-017-06110-5)
Supplement: Supplementary file 1 — Supplementary Figures and Tables [file 41598_2017_6110_MOESM1_ESM.pdf]

Supplementary Figures and Tables for "*High resolution temporal transcriptomics of mouse embryoid body development reveals complex expression dynamics of coding and noncoding loci*"

Brian S. Gloss[1,2], Bethany Signal[1,2], Seth W. Cheetham[3], Franziska Gruhl[4], Dominik Kaczorowski[1], Andrew C. Perkins[5], Marcel E. Dinger[1,2,*]

[1] Garvan Institute of Medical Research, Sydney, Australia

[2] St Vincents Clinical School, Faculty of Medicine, UNSW Australia, Sydney, Australia

[3] The Gurdon Institute and Department of Physiology, Development, and Neuroscience, University of Cambridge, Cambridge, United Kingdom

[4] Center for Integrative Genomics, University of Lausanne, Lausanne, Switzerland

[5] Mater–UQ Research Institute, The University of Queensland, Translational Research Institute, Brisbane, Australia

[*]Corresponding Author: Marcel E. Dinger; tel: +61 2 9355 5860; fax: +61 2 9295 8101, email: m.dinger@garvan.org.au

Supplementary Tables

Supplementary Table S1: Counts of Genes by biotype

Biotype	6 hourly	24 hourly	% increase w 6 hourly	Designation
**antisense**	148	126	17.5%	noncoding_lncRNA
**lincRNA**	265	235	12.8%	noncoding_lncRNA
**processed_transcript**	175	159	10.1%	noncoding_lncRNA
**miRNA**	17	14	21.4%	noncoding_other
**misc_RNA**	3	3	0.0%	noncoding_other
**Mt_rRNA**	2	2	0.0%	noncoding_other
**Mt_tRNA**	3	3	0.0%	noncoding_other
**polymorphic_pseudogene**	5	4	25.0%	noncoding_other
**pseudogene**	292	257	13.6%	noncoding_other
**sense_intronic**	7	7	0.0%	noncoding_other
**sense_overlapping**	2	2	0.0%	noncoding_other
**snoRNA**	40	30	33.3%	noncoding_other
**snRNA**	1	1	0.0%	noncoding_other
**protein_coding**	13336	13036	2.3%	protein_coding
**Total**	**14296**	**13879**		
Novel_MultiExonic_assembled	*1851*	*1174*	*57.7%*	*Novel*

Supplementary Table S2: Periodic Genes

GENCODE_ID	Period	Chr,	Start	End	Strand	Gene Type	Gene Symbol	slRNA
**ENSMUSG00000000826.10**	17.28571429	chr2	181520485	181555133	+	protein_coding	Dnajc5	TRUE
**ENSMUSG00000003847.10**	24.2	chr8	107293470	107379517	+	protein_coding	Nfat5	FALSE
**ENSMUSG00000004187.10**	20.16666667	chr15	76660641	76668196	+	protein_coding	Kifc2	FALSE
**ENSMUSG00000005615.8**	20.16666667	chr16	32430921	32475070	+	protein_coding	Pcyt1a	FALSE
**ENSMUSG00000005682.8**	24.2	chr10	128303335	128321358	+	protein_coding	Pan2	FALSE
**ENSMUSG00000006463.7**	24.2	chr19	4878668	4885397	+	protein_coding	Zdhhc24	FALSE
**ENSMUSG00000008200.8**	24.2	chr2	90745370	90781021	+	protein_coding	Fnbp4	FALSE
**ENSMUSG00000009079.10**	24.2	chr11	5069689	5099266	-	protein_coding	Ewsr1	TRUE
**ENSMUSG00000013483.8**	24.2	chr11	119307768	119345375	+	protein_coding	Card14	FALSE
**ENSMUSG00000016427.7**	30.25	chrX	37187588	37191163	-	protein_coding	Ndufa1	FALSE
**ENSMUSG00000017188.3**	24.2	chr11	101277968	101279114	-	protein_coding	Coa3	FALSE
**ENSMUSG00000018923.7**	24.2	chr11	70451919	70453727	+	protein_coding	Med11	FALSE
**ENSMUSG00000019066.7**	17.28571429	chr9	21907491	21918192	-	protein_coding	Rab3d	FALSE
**ENSMUSG00000020167.8**	20.16666667	chr10	80409514	80433647	-	protein_coding	Tcf3	FALSE
**ENSMUSG00000020530.8**	20.16666667	chr11	84832361	84870817	-	protein_coding	Ggnbp2	TRUE
**ENSMUSG00000020843.9**	24.2	chr11	76406952	76416292	+	protein_coding	Timm22	FALSE
**ENSMUSG00000020894.10**	24.2	chr11	69088490	69092384	+	protein_coding	Vamp2	FALSE
**ENSMUSG00000021124.7**	24.2	chr12	79156017	79172667	-	protein_coding	Vti1b	FALSE
**ENSMUSG00000022805.10**	17.28571429	chr16	38297754	38342143	-	protein_coding	Maats1	FALSE
**ENSMUSG00000024293.9**	15.125	chr18	10566602	10610352	-	protein_coding	Esco1	FALSE
**ENSMUSG00000024959.7**	24.2	chr19	6941861	6951898	+	protein_coding	Bad	FALSE
**ENSMUSG00000025486.10**	30.25	chr7	140863670	140882309	-	protein_coding	Sirt3	FALSE
**ENSMUSG00000025489.4**	20.16666667	chr7	140948963	140950239	-	protein_coding	Ifitm5	FALSE
**ENSMUSG00000025500.5**	20.16666667	chr7	141210043	141214082	-	protein_coding	1600016N20Rik	FALSE
**ENSMUSG00000025868.6**	30.25	chr13	54590207	54591158	+	protein_coding	Higd2a	FALSE
**ENSMUSG00000026034.11**	24.2	chr1	58410189	58424066	-	protein_coding	Clk1	TRUE
**ENSMUSG00000026181.11**	17.28571429	chr16	16896469	16927364	+	protein_coding	Ppm1f	FALSE
**ENSMUSG00000026199.11**	20.16666667	chr1	75192151	75199387	+	protein_coding	Ankzf1	FALSE

**ENSMUSG00000026648.12**	24.2	chr2	3424131	3464130	+	protein_coding	Dclre1c	FALSE
**ENSMUSG00000026792.10**	20.16666667	chr2	32925216	32961614	-	protein_coding	Lrsam1	FALSE
**ENSMUSG00000026864.7**	24.2	chr2	34771970	34776531	+	protein_coding	Hspa5	FALSE
**ENSMUSG00000026977.11**	20.16666667	chr2	60209887	60250676	+	protein_coding	March7	FALSE
**ENSMUSG00000027170.6**	24.2	chr2	104999656	105017080	-	protein_coding	Eif3m	FALSE
**ENSMUSG00000027245.5**	24.2	chr2	121453290	121458672	+	protein_coding	Hypk	FALSE
**ENSMUSG00000027520.9**	20.16666667	chr1	63273265	63314576	+	protein_coding	Zdbf2	FALSE
**ENSMUSG00000027708.8**	30.25	chr3	35892105	35937445	-	protein_coding	Dcun1d1	FALSE
**ENSMUSG00000027782.4**	20.16666667	chr3	69067149	69127113	-	protein_coding	Kpna4	FALSE
**ENSMUSG00000027939.7**	24.2	chr3	90104132	90212017	+	protein_coding	Nup210l	FALSE
**ENSMUSG00000028053.7**	24.2	chr3	88965812	89079373	+	protein_coding	Ash1l	FALSE
**ENSMUSG00000028062.9**	30.25	chr3	88549819	88553074	-	protein_coding	Lamtor2	FALSE
**ENSMUSG00000028790.7**	30.25	chr4	129703164	129742303	-	protein_coding	Khdrbs1	FALSE
**ENSMUSG00000028979.11**	24.2	chr4	148602554	148615499	+	protein_coding	Masp2	FALSE
**ENSMUSG00000028999.9**	15.125	chr5	23787711	23820369	+	protein_coding	Rint1	FALSE
**ENSMUSG00000029068.10**	24.2	chr4	155812489	155824543	+	protein_coding	Ccnl2	FALSE
**ENSMUSG00000029238.8**	24.2	chr5	76212177	76304548	-	protein_coding	Clock	FALSE
**ENSMUSG00000030752.2**	20.16666667	chr7	125444676	125462265	+	protein_coding	Kdm8	FALSE
**ENSMUSG00000031167.10**	24.2	chrX	8138975	8145880	-	protein_coding	Rbm3	FALSE
**ENSMUSG00000031380.10**	24.2	chrX	164373378	164402650	+	protein_coding	Figf	FALSE
**ENSMUSG00000031422.10**	24.2	chrX	136732942	136743690	-	protein_coding	Morf4l2	FALSE
**ENSMUSG00000031433.9**	30.25	chrX	139889511	139998595	-	protein_coding	Rbm41	FALSE
**ENSMUSG00000032350.8**	24.2	chr9	77754535	77794485	+	protein_coding	Gclc	TRUE
**ENSMUSG00000033405.3**	20.16666667	chr14	73519864	73548242	-	protein_coding	Nudt15	TRUE
**ENSMUSG00000033439.6**	24.2	chr3	116581093	116614587	-	protein_coding	Trmt13	FALSE
**ENSMUSG00000034799.10**	24.2	chr8	71624417	71671757	-	protein_coding	Unc13a	FALSE
**ENSMUSG00000035458.8**	20.16666667	chr7	4518305	4524229	-	protein_coding	Tnni3	FALSE
**ENSMUSG00000035476.3**	20.16666667	chrX	85574022	85634469	+	protein_coding	Tab3	FALSE
**ENSMUSG00000035545.7**	24.2	chr7	4137039	4148173	+	protein_coding	Leng8	FALSE
**ENSMUSG00000035637.8**	24.2	chr4	44981395	44990734	+	protein_coding	Grhpr	FALSE
**ENSMUSG00000035666.8**	20.16666667	chr2	28822299	28840360	-	protein_coding	Gtf3c4	TRUE

**ENSMUSG00000035885.4**	30.25	chr19	7215158	7217616	-	protein_coding	Cox8a	FALSE
**ENSMUSG00000036864.8**	20.16666667	chr7	30539134	30552296	-	protein_coding	BC053749	FALSE
**ENSMUSG00000037349.3**	20.16666667	chr19	6993018	6996117	-	protein_coding	Nudt22	FALSE
**ENSMUSG00000037434.7**	20.16666667	chr1	191906767	191913247	+	protein_coding	Slc30a1	TRUE
**ENSMUSG00000037627.9**	24.2	chr15	36009479	36140400	-	protein_coding	Rgs22	FALSE
**ENSMUSG00000038605.11**	20.16666667	chr2	181595218	181599212	-	protein_coding	Samd10	FALSE
**ENSMUSG00000038705.7**	24.2	chr2	181251449	181288035	-	protein_coding	Gmeb2	FALSE
**ENSMUSG00000039463.8**	24.2	chr2	167421712	167477000	+	protein_coding	Slc9a8	FALSE
**ENSMUSG00000039485.6**	30.25	chr10	34297421	34301308	+	protein_coding	Tspyl4	FALSE
**ENSMUSG00000039648.8**	20.16666667	chr2	30185124	30205847	-	protein_coding	Ccbl1	FALSE
**ENSMUSG00000039781.8**	20.16666667	chr11	120064430	120086827	-	protein_coding	Azi1	FALSE
**ENSMUSG00000040429.5**	24.2	chr5	3890581	3893933	-	protein_coding	Mterf	FALSE
**ENSMUSG00000043243.9**	24.2	chr8	71597648	71607936	+	protein_coding	Fam129c	TRUE
**ENSMUSG00000044313.7**	30.25	chr3	101813076	101848959	-	protein_coding	Mab21l3	FALSE
**ENSMUSG00000044966.4**	30.25	chr11	16951375	16954772	+	protein_coding	Fbxo48	FALSE
**ENSMUSG00000046909.9**	30.25	chr11	80136678	80142175	-	protein_coding	Tefm	FALSE
**ENSMUSG00000047182.5**	20.16666667	chr5	137643032	137645714	-	protein_coding	Irs3	FALSE
**ENSMUSG00000047909.5**	24.2	chr2	11777876	11790325	+	protein_coding	Ankrd16	FALSE
**ENSMUSG00000048106.4**	15.125	chr3	19894886	19895962	+	protein_coding	4632415L05Rik	FALSE
**ENSMUSG00000049164.6**	20.16666667	chr19	40894705	40917947	+	protein_coding	Zfp518a	TRUE
**ENSMUSG00000049470.7**	20.16666667	chr11	53350833	53421830	+	protein_coding	Aff4	FALSE
**ENSMUSG00000050088.9**	20.16666667	chr17	14943184	14959570	+	protein_coding	1600012H06Rik	FALSE
**ENSMUSG00000050357.8**	24.2	chr8	105690906	105698178	+	protein_coding	Rltpr	FALSE
**ENSMUSG00000050856.10**	30.25	chr5	108433244	108434448	-	protein_coding	Atp5k	FALSE
**ENSMUSG00000051278.8**	24.2	chr3	127553489	127618021	+	protein_coding	4930422G04Rik	FALSE
**ENSMUSG00000053964.10**	24.2	chr7	28833975	28841703	+	protein_coding	Lgals4	FALSE
**ENSMUSG00000054051.6**	24.2	chr14	32513521	32580989	+	protein_coding	Ercc6	FALSE
**ENSMUSG00000054099.5**	24.2	chr5	8422850	8454790	+	protein_coding	Slc25a40	TRUE
**ENSMUSG00000055833.8**	15.125	chr1	191890233	191907527	-	protein_coding	1700034H15Rik	FALSE
**ENSMUSG00000057262.1**	24.2	chr15	52477614	52478228	-	protein_coding	Gm10020	FALSE
**ENSMUSG00000060032.4**	30.25	chr6	136808248	136810074	+	protein_coding	H2afj	TRUE

**ENSMUSG00000063108.8**	20.16666667	chr9	20432972	20460162	-	protein_coding	Zfp26	FALSE
**ENSMUSG00000066057.6**	24.2	chr17	94759944	94834799	-	lincRNA	Gm1976	FALSE
**ENSMUSG00000067833.2**	30.25	chr2	156392491	156392829	-	protein_coding	2900097C17Rik	FALSE
**ENSMUSG00000068221.4**	20.16666667	chr15	78913919	78919517	+	protein_coding	Pdxp	FALSE
**ENSMUSG00000068917.6**	20.16666667	chr3	89164795	89176921	+	protein_coding	Clk2	TRUE
**ENSMUSG00000069890.3**	24.2	chr11	49586641	49588462	-	pseudogene	1700024J04Rik	FALSE
**ENSMUSG00000070282.6**	30.25	chr9	109827943	109832706	-	pseudogene	3000002C10Rik	FALSE
**ENSMUSG00000070343.2**	24.2	chr3	146838775	146839365	-	protein_coding	Gm10288	FALSE
**ENSMUSG00000070490.4**	30.25	chr3	52612835	52613836	+	pseudogene	Gm10293	FALSE
**ENSMUSG00000070808.3**	20.16666667	chr7	15971262	15999495	-	protein_coding	Gltscr1	FALSE
**ENSMUSG00000071037.5**	17.28571429	chr17	85090726	85458139	+	protein_coding	Camkmt	FALSE
**ENSMUSG00000071528.3**	24.2	chr19	47083471	47090625	-	protein_coding	Usmg5	FALSE
**ENSMUSG00000071632.4**	20.16666667	chr16	18836578	18840158	+	protein_coding	2510002D24Rik	FALSE
**ENSMUSG00000071796.7**	24.2	chr8	20262613	20297546	-	pseudogene	6820431F20Rik	FALSE
**ENSMUSG00000072294.4**	24.2	chr14	99874486	100149764	-	protein_coding	Klf12	FALSE
**ENSMUSG00000072762.3**	20.16666667	chr5	109735990	109751886	-	protein_coding	4930522L14Rik	FALSE
**ENSMUSG00000074925.3**	20.16666667	chr19	23687400	23721129	+	protein_coding	Ptar1	FALSE
**ENSMUSG00000075376.4**	17.28571429	chr2	37370069	37422903	-	protein_coding	Rc3h2	FALSE
**ENSMUSG00000075463.3**	24.2	chr14	122913030	122937469	+	processed_transcript	4930594M22Rik	FALSE
**ENSMUSG00000078162.4**	17.28571429	chr3	142962561	142963792	+	pseudogene	Gm2574	FALSE
**ENSMUSG00000078619.4**	17.28571429	chr11	106263179	106272972	-	protein_coding	Smarcd2	TRUE
**ENSMUSG00000078651.2**	24.2	chr11	101325063	101329702	+	protein_coding	Aoc2	FALSE
**ENSMUSG00000078919.4**	24.2	chr2	168209048	168230591	-	protein_coding	Dpm1	FALSE
**ENSMUSG00000079065.2**	24.2	chr5	104508352	104522383	+	protein_coding	BC005561	FALSE
**ENSMUSG00000079509.4**	20.16666667	chrX	94074631	94123702	-	protein_coding	Zfx	TRUE
**ENSMUSG00000080727.2**	20.16666667	chr3	95871522	95889093	+	protein_coding	C920021L13Rik	FALSE
**ENSMUSG00000081739.3**	24.2	chrX	128261411	128267924	+	pseudogene	Mdm4-ps	FALSE
**ENSMUSG00000082016.2**	17.28571429	chr7	27468890	27470557	-	pseudogene	Pgam1-ps2	FALSE
**ENSMUSG00000083816.2**	24.2	chr4	132884545	132886626	+	pseudogene	Gm13033	FALSE
**ENSMUSG00000084846.1**	24.2	chr3	94994410	94997350	-	processed_transcript	A730011C13Rik	FALSE
**ENSMUSG00000084892.1**	24.2	chr2	30306067	30323986	-	processed_transcript	Gm14471	FALSE

**ENSMUSG00000084899.1**	24.2	chr10	86692119	86693095	-	processed_transcript	Gm15344	FALSE
**ENSMUSG00000086904.1**	24.2	chr2	33978246	33980324	+	lincRNA	Gm13404	TRUE
**ENSMUSG00000087060.1**	24.2	chr11	16935154	16951282	-	processed_transcript	2810442I21Rik	FALSE
**ENSMUSG00000087153.1**	24.2	chr8	19682268	19698275	+	protein_coding	Gm6483	FALSE
**ENSMUSG00000087165.1**	24.2	chr11	57795499	57801417	-	processed_transcript	2010001A14Rik	FALSE
**ENSMUSG00000087373.1**	17.28571429	chr11	88059066	88067476	+	lincRNA	Gm15892	FALSE
**ENSMUSG00000089788.1**	24.2	chr1	178321108	178324171	-	processed_transcript	Gm16586	TRUE
**ENSMUSG00000090086.1**	24.2	chr5	123133723	123141666	-	lincRNA	AI480526	TRUE
**ENSMUSG00000090330.1**	20.16666667	chr7	25228687	25230257	+	protein_coding	9130221H12Rik	FALSE
**ENSMUSG00000090952.2**	20.16666667	chr17	33759992	33761870	+	processed_transcript	Gm17251	FALSE
**ENSMUSG00000092275.1**	24.2	chr5	33293614	33311150	-	lincRNA	Gm20465	TRUE
**ENSMUSG00000096401.1**	24.2	chr8	20136455	20150494	+	protein_coding	Gm21811	FALSE
**ENSMUSG00000097392.1**	24.2	chr5	104525735	104554211	+	lincRNA	D930016D06Rik	FALSE
**ENSMUSG00000097743.1**	20.16666667	chr14	56696280	56701268	-	lincRNA	Gm16973	TRUE
**ENSMUSG00000098161.1**	24.2	chr9	118404097	118406099	-	lincRNA	Gm26975	FALSE
**ENSMUSG00000098912.1**	24.2	chr3	88782943	88832547	-	processed_transcript	RP23-199B2.4	FALSE

Supplementary Figures and Legends

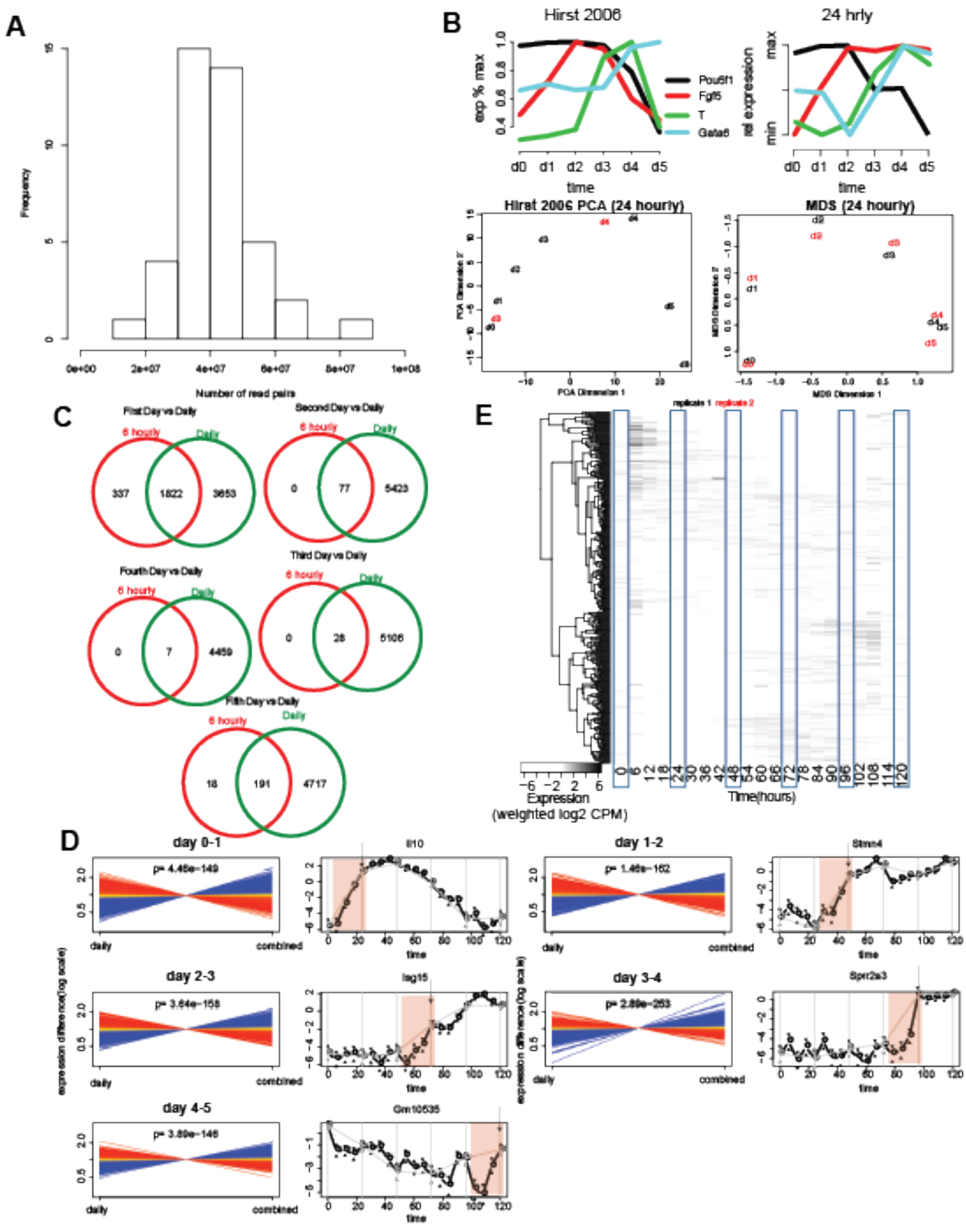

Supplementary Figure S1: Global evaluation of high-resolution transcriptomic data. (**A)** Histogram of mapped read number distribution per sample (pooled from biological replicates). (**B)** Comparison of expression levels and principle components analysis measured 24 hourly between this study and Hirst 2006 **(C)** Evidence of differential expression within one 24 hour period vs. any change across all 24hourly times (p <0.0001). **(D)** Comparing whether the 24 hourly measures "summarize" that 24 hour window by comparing mean expression for that window with the 24 hour time point, a colour gradient was assigned by ranked expression levels in the daily measure. P-value is from a paired Wilcoxon rank-sum test. **(E)** Greyscale heatmap of expression levels for genes only expressed outside of 24 hourly time points, clustered by expression pattern.

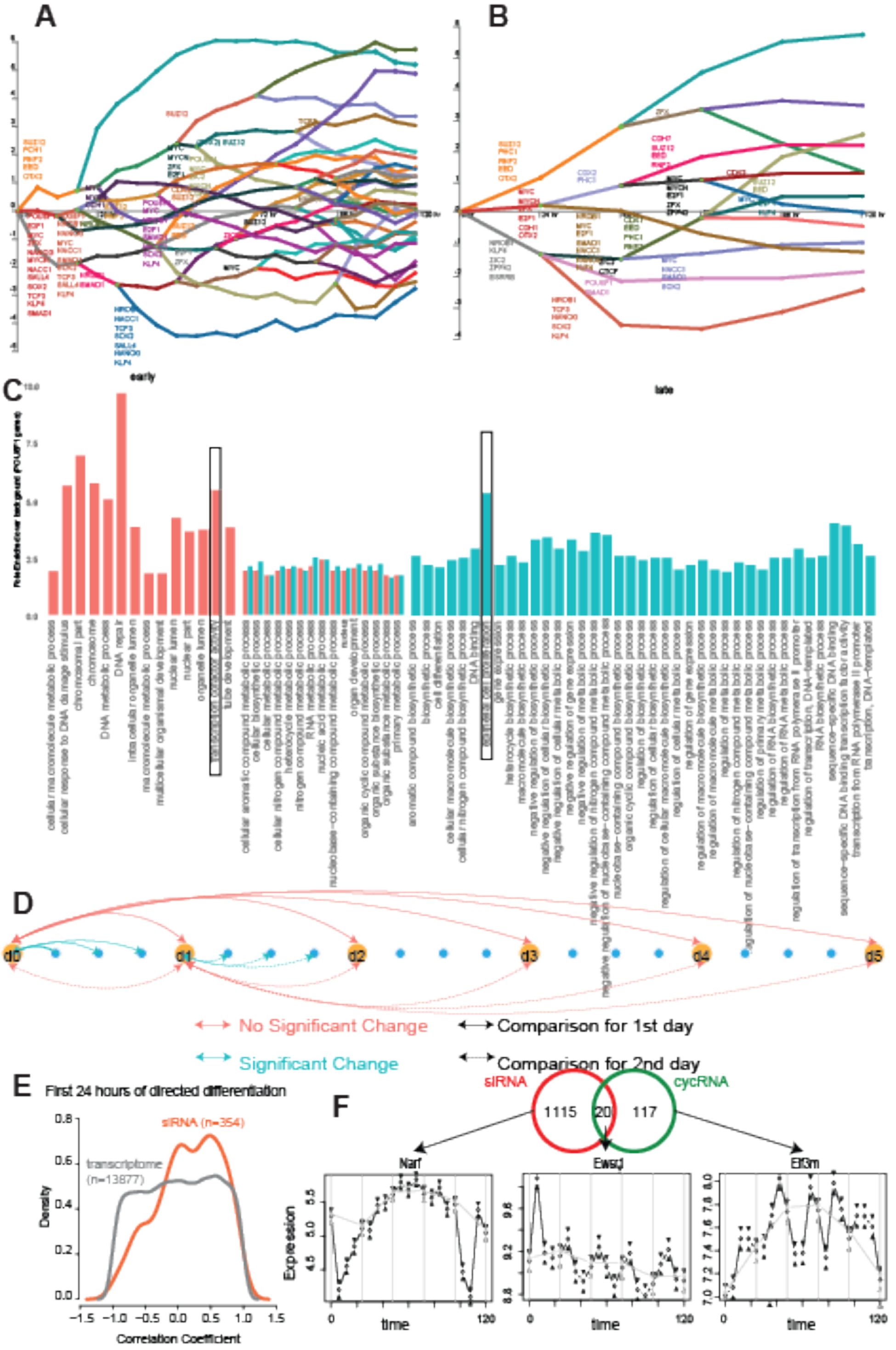

Supplementary Figure S2: Highlighting unique knowledge gained from increased temporal resolution. (A &B) Fully annotated DREM schematic of estimated TF activity of key ESC related TFs at 6hourly (A) vs. 24 hourly (B). Relative circle sizes are proportional to the spread of gene expression levels corresponding to that point. line colors are assigned by branch and are not comparable between panels. Red and blue boxes pertain to branch points of interest **(C)** GO term enrichment (adjusted $p<0.05$) for genes corresponding branch points designated as early (co-observed with change in POU expression) and late (observed after POU5f1 Expression changes) highlighted by the blue boxes in Figure 2A. Black boxes represent similar terms identified in Figure 2D. (**D**) Schematic of differential expression analysis design used to identify slRNAs. (**E**) Correlation of slRNA expression in [21]. **(F)** Comparison of slRNAs and cycRNAs. Venn diagram of the overlap observed and examples from each class.

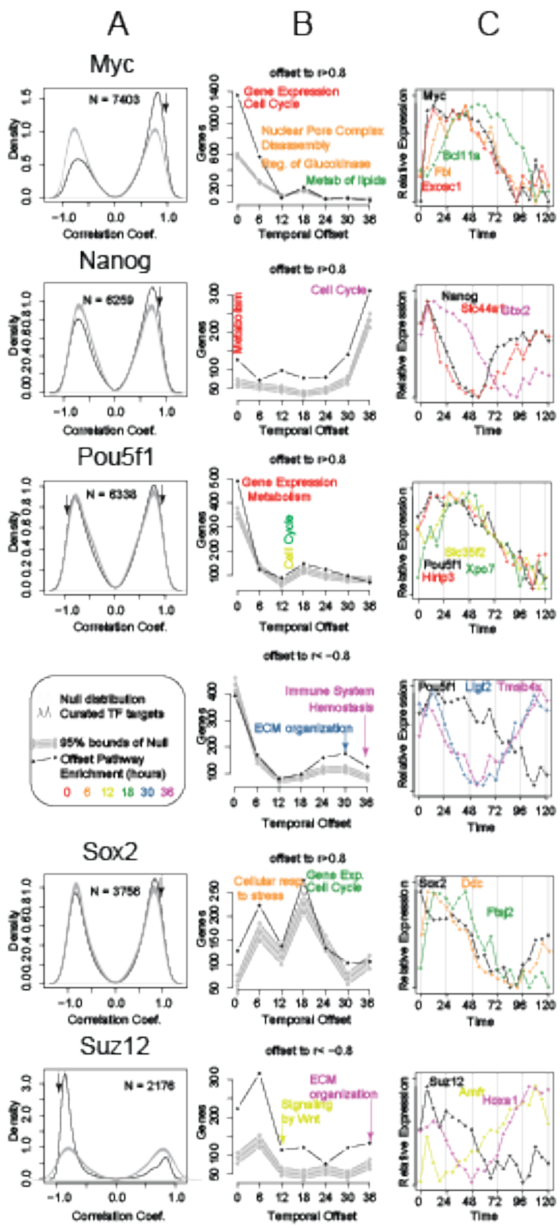

Supplementary Figure S3: Temporal offsets in transcription factor (TF)- target gene expression. (**A)** Curated TF/gene targets were downloaded from chea (http://amp.pharm.mssm.edu/lib/chea.jsp) for Myc, Nanog, Pou5f1, Sox2 and Suz12 (N=number of target genes). Expression of target genes were tested for correlation with their TF at different temporal offsets (0-36 hours) and compared to 500 random selections of the same number of genes (Null). Where absolute correlations of predicted targets exceeded the null distribution (arrow), (**B)** the number of genes achieving a maximal absolute correlation of >0.8 and the offset required to reach these maxima was plotted against the 5th and 95thquantiles of the same results from the null distribution. Where the number of target genes exceeded the null distribution, the lists of genes in each offset were tested for enrichment of Reactome pathways relative to the total predicted target list (enrichment). **(C)** Example expression patterns of genes displaying these attributes were plotted.

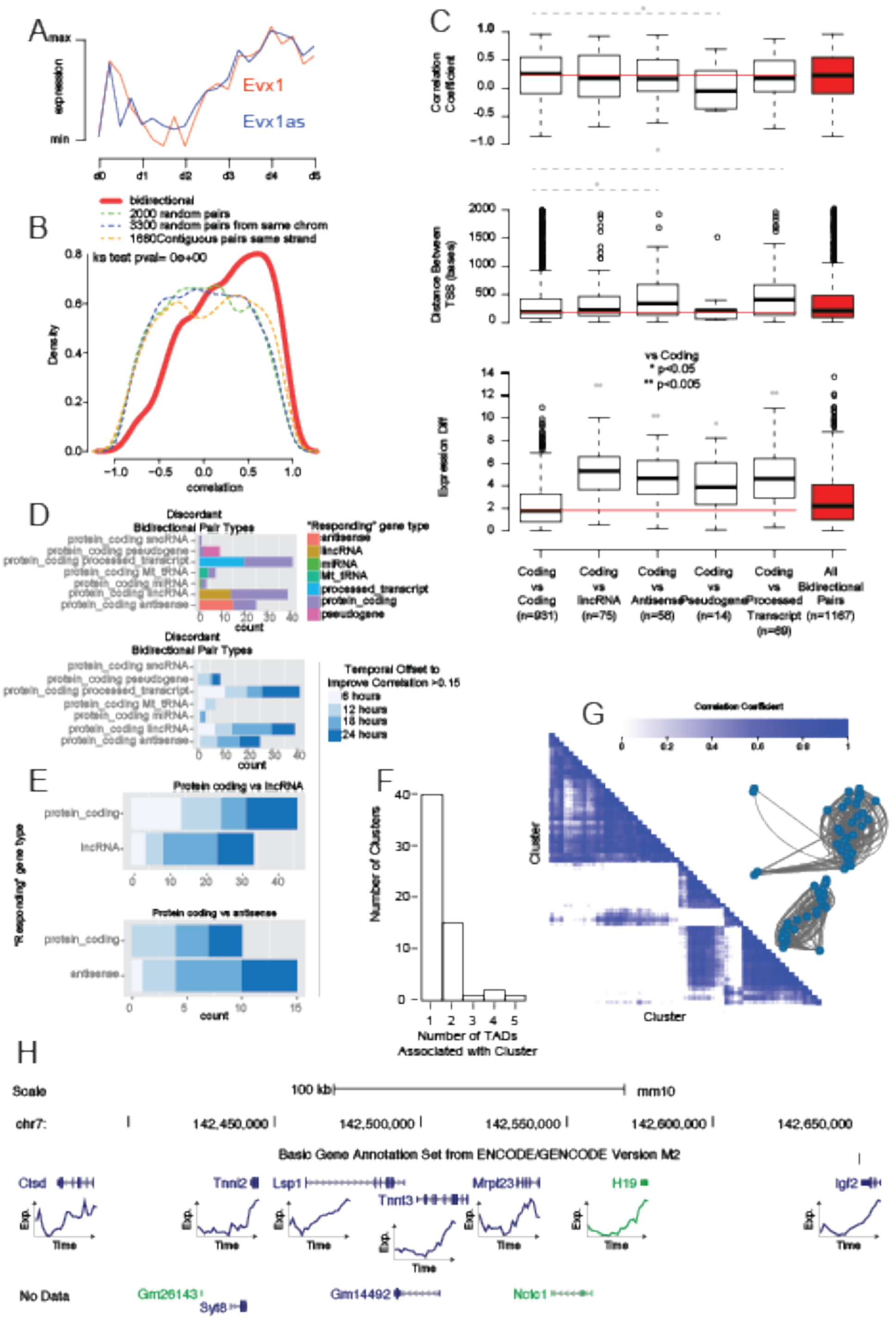

Supplementary Figure S4: Bidirectional and co-expression analysis of mouse ES development. (A) Expression profile of EVX1 and its antisense (and positively correlated) transcript EVX1AS- the peak at 6-18 hours has not been observed previously. **(B)** Distribution of correlation coefficients of bidirectional gene pairs (red) compared to similar numbers of randomly chosen genes pairs, randomly chosen genes from the same chromosome and, randomly selected neighbouring genes (dotted lines). ks=Kolmogorov–Smirnov test (bidirectional vs. random neighbouring gene pairs). **(C)** Characteristics of bidirectional gene pairs (Correlation coefficient, Distance between TSS and Difference of median expression (log scale) based on annotated gene-biotype (p value calculated using the Mann-Whitney test. **(D)** Counts of bidirectional gene pairs of differing biotypes achieving an improved correlation coefficient of >0.15 (to at lease 0.25) over that at time zero, colored by the biotype of the "following" gene or by the temporal offset required to achieve the improvement. **(E)** Comparison of responding gene biotype to the temporal offset for lincRNA and antisense biotypes. **(F)** Number of topological associated domains (TADS, HindIII data mapped to mm10 using liftOver from mm9) associated with each co-expressed gene cluster. **(G)** Clustering of co-regulated gene clusters visualized by network diagram and hierarchical clustering of the correlation matrix showing two distinct groups. **(H)** The imprinted H19/IGF2 cluster identified as a co-expressed gene cluster with gene expression data for measured genes. Some genes did not have expression data (no data).

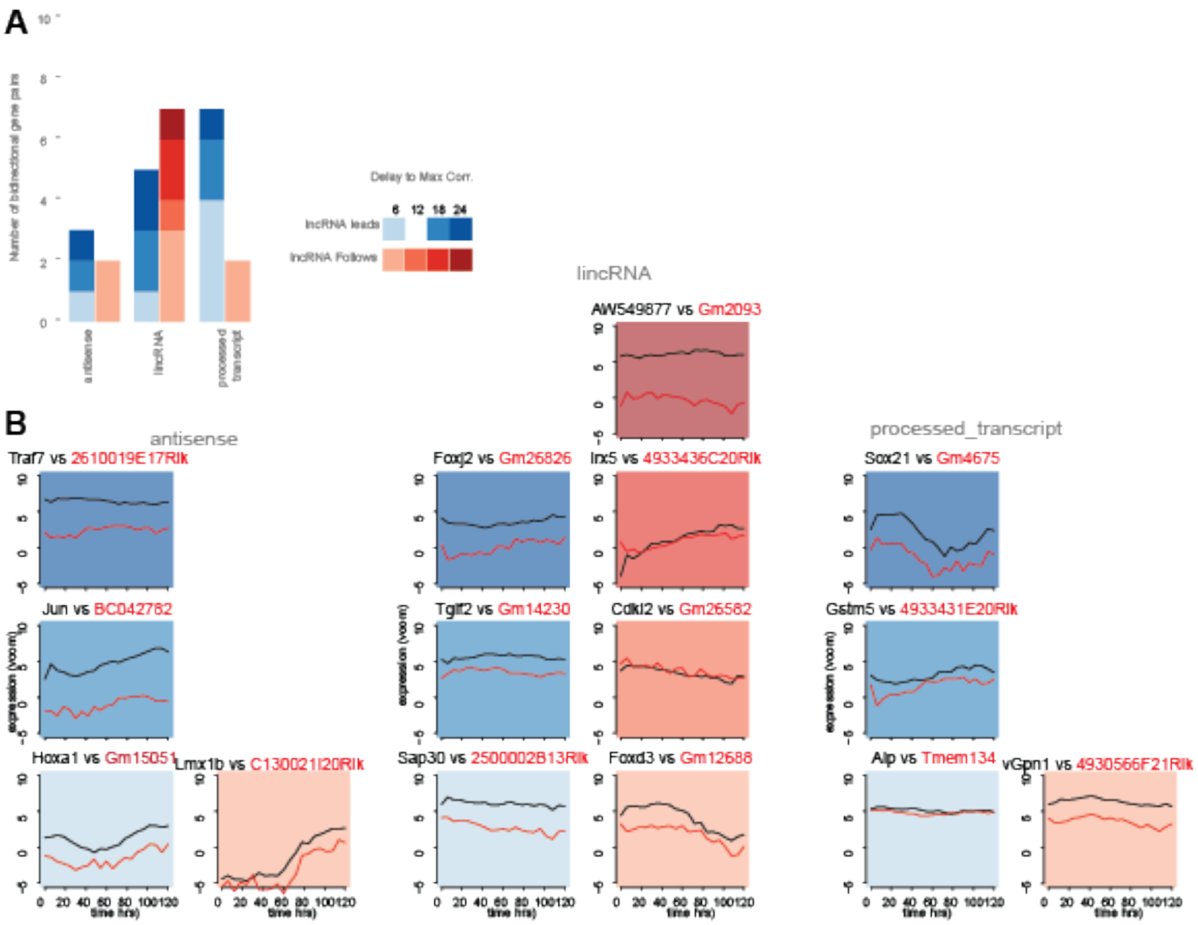

Supplementary Figure S5: Temporal relationships of highly correlated coding-noncoding bidirectional pairs. (A) Bar chart of the temporal offset required to reach a maximum correlation >0.8 and whether the noncoding gene preceded the protein coding gene or vice versa. **(B)** Example gene expression profiles of bidirectional paired gene over the time course. Gene profiles are arranged and colored as the bar chart.

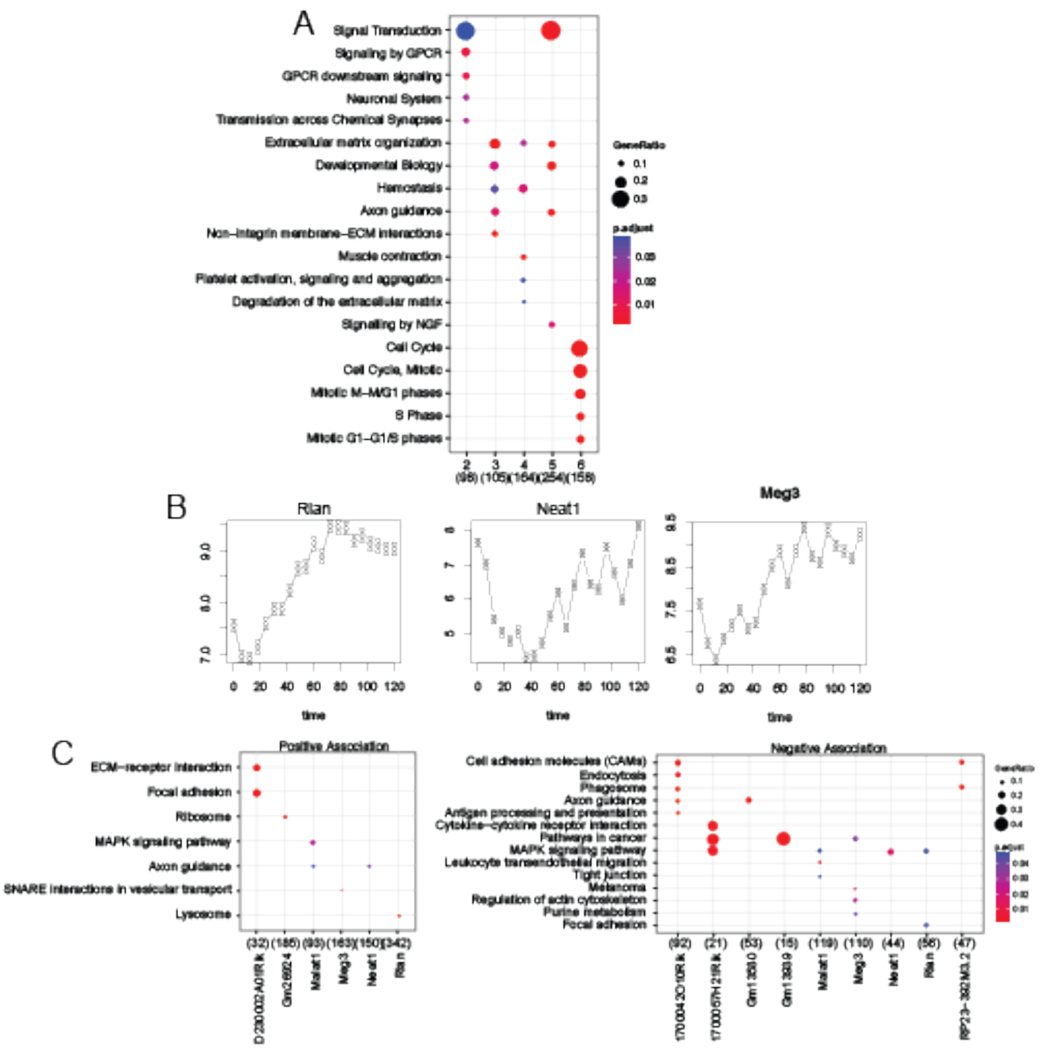

Supplementary Figure S6: LncRNAs and their role in ES development. (A) Reactome pathway enrichment for 5/6 k-means clusters of time-dependent protein coding genes. (**B)** Expression profiles for characterized lncRNAs described in text. (**C)** Reactome pathway enrichment for putative gene targets positively or negatively associated with candidate lncRNAs (top 4 pathways, enrichment adj. pval. <0.05)

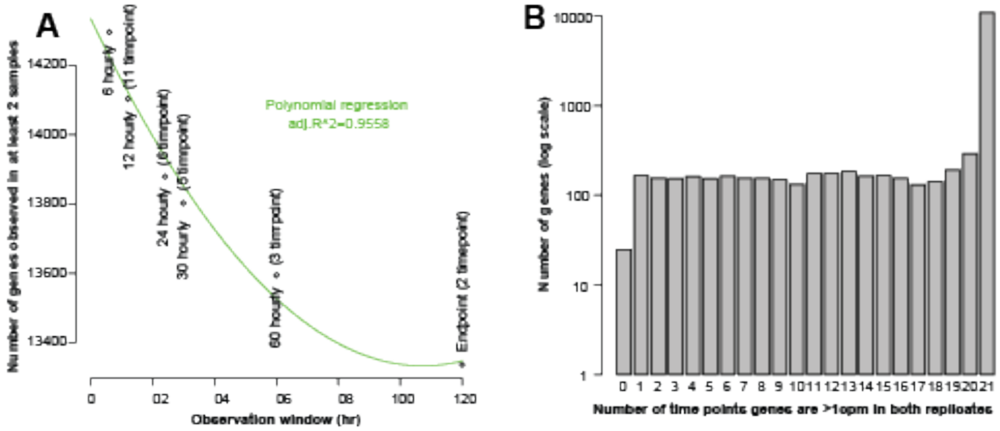

Supplementary Figure S7: (A) The impact of increasing temporal resolution on the number of genes observed to be expressed. **(B)** The number of conditions in which each gene observed is expressed above background in both replicates across the time course. ~150 new genes are observed at a single time point.
